# The collapse of gene complement following whole genome duplication

**DOI:** 10.1186/1471-2164-11-313

**Published:** 2010-05-19

**Authors:** David Sankoff, Chunfang Zheng, Qian Zhu

**Affiliations:** 1 Department of Mathematics and Statistics, University of Ottawa, Ottawa, K1N 6N5 Canada; 2 Département d'informatique et de recherche opérationnelle, Université de Montréal, Montréal, Québec, H3C 3J7, Canada; 3 Department of Computer Science, Princeton University, Princeton, NJ, 08544 USA

## Abstract

**Background:**

Genome amplification through duplication or proliferation of transposable elements has its counterpart in genome reduction, by elimination of DNA or by gene inactivation. Whether loss is primarily due to excision of random length DNA fragments or the inactivation of one gene at a time is controversial. Reduction after whole genome duplication (WGD) represents an inexorable collapse in gene complement.

**Results:**

We compare fifteen genomes descending from six eukaryotic WGD events 20-450 Mya. We characterize the collapse over time through the distribution of runs of reduced paralog pairs in duplicated segments. Descendant genomes of the same WGD event behave as replicates. Choice of paralog pairs to be reduced is random except for some resistant regions of contiguous pairs. For those paralog pairs that are reduced, conserved copies tend to concentrate on one chromosome.

**Conclusions:**

Both the contiguous regions of reduction-resistant pairs and the concentration of runs of single copy genes on a single chromosome are evidence of transcriptional co-regulation, dosage sensitivity or other functional interaction constraining the reduction process. These constraints and their evolution over time show a consistent pattern across evolutionary domains and a highly reproducible pattern, as replicates, for the several descendants of a single WGD.

## Background

Following an episode of whole genome doubling (WGD), gene duplicates are lost at an initially high rate through processes such as epigenetic silencing, pseudogenization, and deletion of chromosomal segments containing one or more genes, while intra- and inter-chromosomal rearrangement mechanisms redistribute chromosomal segments both large and small across the genome. The genome of a present-day descendant can be largely decomposed into a set of duplicated DNA segments dispersed among the chromosomes, with all the duplicate pairs of genes exhibiting a similar degree of sequence divergence, and with segments containing only single-copy genes interspersed among them. The present paper proposes a resolution of the controversy as to what extent paralog reduction is a gene-by-gene process [1], targeting redundant copies at random points throughout the genome, whose loss restores, or at least does not perturb, functional balance; and to what extent it is a consequence of largely random elimination of excess DNA [2]. These two processes may often coincide, since 1.) the actual excision of critical exons of a single gene is one of the ways a gene can be lost, along with various other suppression and silencing mechanisms leading to pseudogenization, and 2.) even if two or more adjacent genes are lost at the same time, this may be the result of of their regulatory interaction, dosage compensation [3], epigenetically marked homeolog preference [4] or functional buffering [5], rather than the deletion of a DNA fragment.

The key evidence in studying the pattern of gene losses across the genome has been the distribution of the length of runs of single-copy genes [6,7]. The chief methodological difficulty has been the increasing rate of disruption of these runs over time by chromosomal rearrangement.

The evidence in this paper comes from six distinct WGD events across the eukaryotic spectrum, including three in which we examine multiple independent descendants. The time scale ranges from 20 My to 450 My. We argue that the pattern of gene loss across the genome must be studied at two levels. At the higher level, where we test whether gene loss events are scattered randomly throughout the genome, we ask how duplicate pairs of paralogs on homeologous chromosomes are chosen to be reduced to single-copy. Because loss of *both *copies is likely to result in diminished viability in at least some natural contexts, it makes more sense to narrow the null hypothesis so that an independent loss process affects entire paralog pairs, rather than all genes including single-copy ones. At the lower level, where we test whether more than one gene tends to be lost at a time, we ask whether the "survivors" of paralog pair reduction, rather than being divided (*fractionated *or *interleaved*) randomly between the two homeologous chromosomes, are located disproportionately on one of them, as has been demonstrated for the particular case of *Arabidopsis *[4]. We seek answers to both questions by identifying all pairs of single-copy regions where strict criteria allow us to be fairly sure that the two regions were originally paralogous and that neither has been been disrupted by rearrangements swapping out some genes or introducing external genes. These are our *analytical units *(AU), similar to the *consolidated *regions in [8]. We develop ways of visualizing the level of statistical significance of fractionation in all AU containing a given number *s *of single-copy genes, both for all *s *simultaneously, and separately for each *s *as a way of fitting a geometric distribution of deletion lengths.

**Figure 1:**
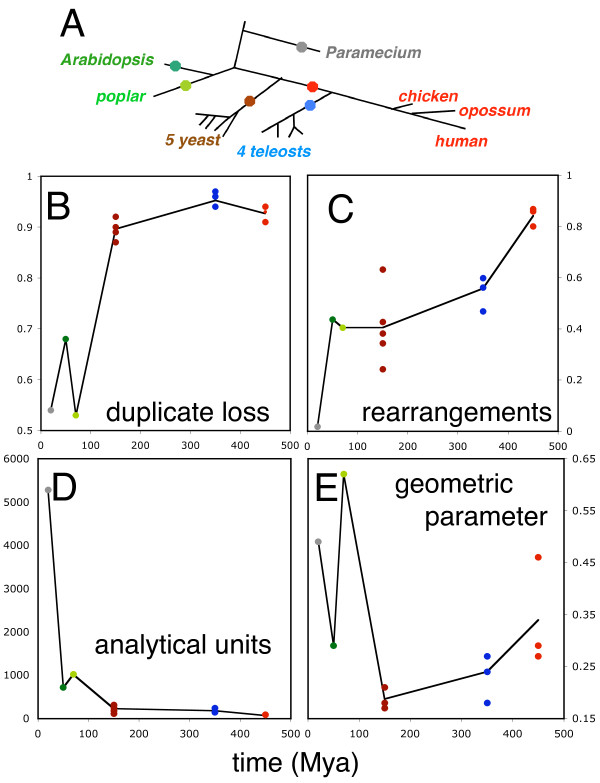
**Parameters of genome collapse**. (A) Locating WGD events on eukaryotic phylogeny. The WGD events (shown as colored dots) are assigned the following dates: 20, 50, 70, 150, 350, 450 Mya for *Paramecium, Arabidopsis, Populous*, yeast, teleosts, and higher vertebrates. (B) Proportion of duplicate pairs reduced, *c *= 2*m*/(*n *+ *m*), where *m *is the number of single-copy genes deriving from reduced pairs and *n *is the total number of genes in the genome. (C) "Halving" distance; minimum number of genome rearrangements *d *necessary to a convert any inferred ancestral tetraploid to present-day genome, normalized, *D *= 2*d*/(*n *- *m*). (D) Number of analytical units (AU). (E) Parameter of geometric distribution fitted to distribution of AU lengths *m *> 0. In all these figures, multiple descendants of single WGD events (shown as vertically aligned same-color dots) have similar parameters, indicating a conserved gene-loss dynamic across these descendants.

The present-day genomes we analyze (and WGD events in their ancestry) are: *Paramecium tetraurelia *(most recent of four or more WGD events in its ancestry [9]); *Saccharomyces cerevisiae*, *Saccharomyces bayanus*, *Candida glabrata*, *Naumovia castelli *and *Vanderwaltozyma polyspora *(yeast doubling event discovered by Wolfe and colleagues [10]); *Populus trichocarpa *(WGD event in the Salicaceae) [11]; *Arabidopsis thaliana *(most recent WGD [12]); *Tetraodon nigroviridis*, *Takifugu rubripes*, *Oryzias latipes*, *Gasterosteus aculeatus *(teleost WGD [13,14]) and chicken, opossum and human (most recent vertebrate WGD [15]). The phylogenetic diversity of this sample is illustrated in Figure 1A. Although there are likely multiple WGD in each of these lineages, except yeast, we focus on the most recent WGD in each case, using curated paralogies where available (yeast, *Arabidopsis, Paramecium*) and protein alignment scores elsewhere to identify pertinent paralogies.

From our data, the paralog pair reduction events are consistent with both hypotheses, the random choice of pairs across the genome, and the random deletion of DNA fragments, as long as these fragment lengths are distributed as a negative exponential or geometric distribution. There is a major exception, in that some groups of adjacent paralog pairs seem resistant to reduction.

The closer study of the partition of single-copy genes to the two chromosomes within the AU reveals a distinct tendency for adjacent genes to be located on the same chromosome. This results in longer runs of single-copy genes on one of the chromosomes than would be expected in a random partition model.

Over time this pattern of fractionation breaks down, particularly for shorter AU while some longer runs of single-copy genes survive, presumably under selective pressure at some level.

In establishing these trends, we also discover great statistical regularity in the process of gene loss across the eukaryotes and especially among the independent descendants of a single WGD.

## Results and Discussion

### Dynamics of genome collapse

Figures 1B-1E summarize the gross statistical analysis of the fifteen genomes, descendants of the six WGD events. Figure 1B shows how the loss of paralogs roughly reflects the age of the WGD, at least over the first 150 My. After this period, a residual 5-10% of unreduced paralogies can be attributed partly to functional divergence of the two genes [14], although WGD paralogs have been shown to differentiate functionally less than do duplicate gene pairs originating through other mechanisms [16]. Figure 1C measures the monotonic increase in rearrangement distance between the present-day genome and the closest possible tetraploid ancestor, calculated by a "genome halving" algorithm [17]. Of particular interest in these two graphs is the relatively tight clustering of points representing genomes descending from the same WGD. (The disproportionately rearranged yeast genome is that of *C. glabrata*.)

### Analytical units

**Figure 2:**
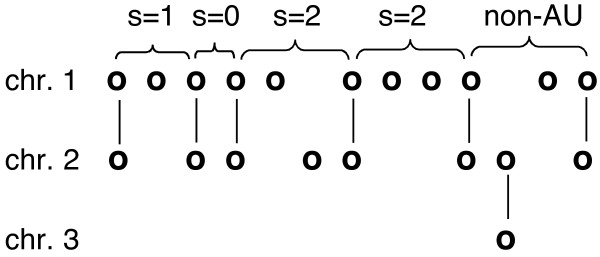
**Analytical units (AU)**. The number of single copy genes *s *bounded by pairs of duplicates on chromosomes 1 and 2 is the sum of those on chromosome 1 and chromosome 2. The last two pairs of duplicates on chromosomes 1 and 2 do not bound an AU because one of the genes between them has a paralog on chromosome 3.

To mitigate the effects of genome rearrangement in truncating runs of single-copy genes or artifactually creating such runs, we focus on "analytical units" (AU), each consisting of a set of single-copy genes bounded at both ends by a pair of duplicate genes, in parallel orientation, on the same two chromosomes, as depicted in Figure 2. The requirements on the duplicate genes assures us that, with a few coincidental exceptions, the intervening single-copy genes, on one or the other of the two chromosomes, arose through the loss of one copy from a corresponding position on the other chromosome and that no rearrangement has interchanged material from outside the AU with material inside it.

Thus, in contrast to much previous work, but in line with recent *Arabidopsis *research [4,12], we consider AUs containing single copies on two chromosomes instead of just one because the fundamental constraint on deleting a gene is whether its paralog has already been deleted. Thus the choice of gene to delete is really two simultaneous choices, the first which pair of paralogs to reduce, and the second within a pair to be reduced, which of the two copies to eliminate. Are duplicate pairs to be reduced chosen at random across the genome? Are the single gene copies within an AU conserved indifferently on both chromosomes, or do they tend to fall on only one of the chromosomes?

### Preliminary overview of the analysis of AUs

For the first question, under a model of random choice of paralog pair to reduce, the number *s *of single copy genes within an AU should be approximately geometrically distributed as *N*(*s*) = *N*(0)(1 - *p*)^*s*^*p*. The parameter *p *should be a decreasing function of the number of gene losses, and an increasing function of the number of rearrangements, as in Figure 1E. Indeed, as evolution proceeds, the number of AU and their average lengths *s *should increase over time, until there are relatively few paralogy pairs left to reduce while the number of genomic rearrangements continues to increase, disrupting AUs. These conflicting processes affecting the number of AUs result in the pattern in Figure 1D. Again, in both of these figures, there is a striking tendency for genomes descending from the same WGD event to cluster together, indicating a common genomic dynamic determined by the size and structure of the initial doubled genome and the common inherited evolutionary tendencies arising from DNA repair mechanisms, generation time, and other factors.

Under a model of DNA excision, we can postulate a distribution of DNA fragment lengths in base pair units at each deletion, the simplest being an negative exponential. At the gene level this translates roughly into a geometric distribution. As deletions accumulate, some of the AUs become longer by the accumulation of fragments, while at the same time new, relatively short, AUs are created.

### Distribution of lengths of runs of reduced pairs

**Figure 3:**
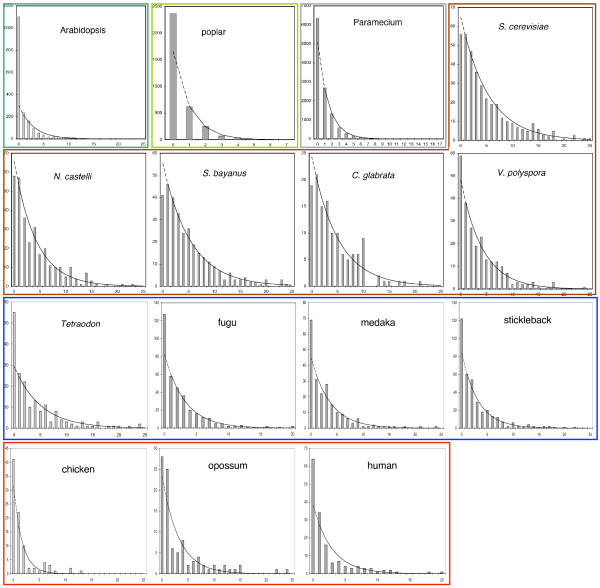
**Distribution of the size *s *(number of single-copy genes) in AUs**. Colored outlines include genomes descending from a single WGD. Solid curve: geometric fit (minimum chi-square) to data for *s *> 0 only. Dotted segment: extrapolation to *s *= 0.

For each of the fifteen genomes studied, Figure 3 compares *N*(*s*), the observed frequency of occurrence of AUs of length *s *with a geometric distribution fitted by minimizing chi-square over all values of the parameters *N*(1) and *p*. The value of *N*(0) was not used in this estimation. Instead, we extrapolated the geometric distribution, predicting  by . It can be seen that there is no systematic deviation from a geometric law for *s *≥ 1. Aside from the case *s *= 0, this is consistent with paralogy reduction where duplicate pairs are chosen randomly. But it does not exclude random DNA fragment elimination, which can also produce a geometric distribution, as we will explain in the next two sections.

One unexpected observation is the under-prediction of *N*(0) by the geometric distribution model. In the present-day genomes descending from all the WGD events, except that for yeast, we find a larger number of duplicate pairs immediately adjacent to each other on both chromosomes, i.e., *s *= 0, than expected. As can be seen in Figure 1B, yeast has progressed the furthest in the loss of duplicate genes, and this may account for why it no longer retains this pattern.

### Concentration of single-copy genes

For the second question, which paralog to conserve when a paralog pair is reduced, if the genes are affected independently, we can calculate the probability that exactly *q *out of the *s *single-copy genes in an AU occur on the same chromosome and *s *- *q *on the other is *b*(*s, q*) + *b*(*s*, *s *- *q*), for , where *b *is the binomial distribution. The cumulative probability that *q *or fewer single-copy genes, out of *s*, will appear on the same chromosome [18] is . (The summation only goes to  in order to treat the two homeologous regions symmetrically.) This suggests a summary statistic measuring the degree of concentration of the single copy genes within the AU on one chromosome or the other, , the cumulative empirical frequency, where *F *(*s*, *q*) is the proportion of AUs of size *s *containing *q *of the single-copy genes in the AU on either chromosome and *s *- *q *on the other. Note that these statistics pertain to individual AUs and not to entire chromosomes. Because the genomes have been rearranged, heavily in some cases, there is generally no way to reconstruct which "side" of an AU was on which homeologous chromosome after the WGD.

**Figure 4:**
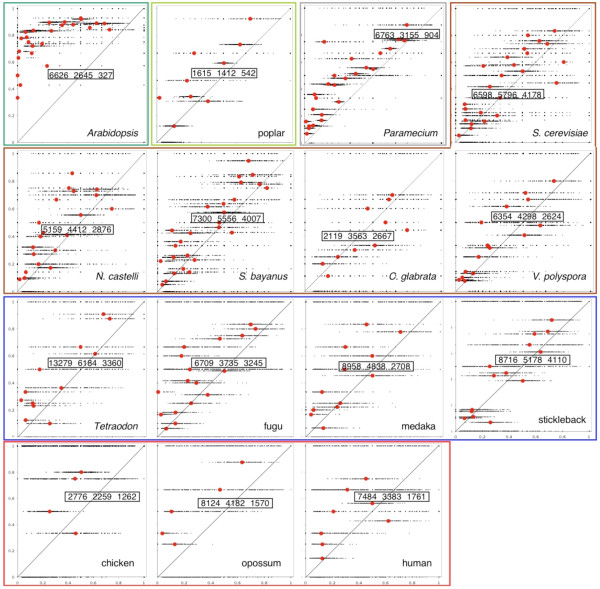
**Cumulative probabilities for concentration of single-copy genes in comparable AUs in real (vertical axis) and simulated (horizontal axis) genomes**. Simulated genomes are derived from a reconstructed ancestral duplicated genome by random paralog reduction, random gene deletion within paralog pairs, and random rearrangements. Boxed "above/on/below diagonal" figures reproduced in Table 1. Almost all real genomes show significantly more asymmetry or concentration of single-copy genes than the simulated genomes for AUs of the same size.

For each of the fifteen genomes, Figure 4 compares the cumulative frequency of *s *in the AUs of the data genome with simulated data. We ran 200 simulations of the evolution of the genome from the original tetraploid containing *n *+ *m *genes in all, with random choice of *m *paralog pairs to reduce, and random choice of chromosome on which to conserve single copies. I.e., each of the two copies had a 50% chance of surviving. We calculated  for AUs of each size *s *with the corresponding cumulative frequency for AUs of that size in each of the simulated genomes. The number of rearrangements *d *to simulate was inferred through genome halving [17] and the number of paralog reductions was *m*. Note that even if the simulations are biased in the total number of AUs or the number of AUs of a given size, there is no bias to be expected for the concentration of genes on one chromosome or the other since we will be comparing only AUs of the same size.

It can be seen that in the overwhelming majority of cases, the real genomes exhibit greater concentration of genes on one or the other chromosome ("biased fractionation" [8]) than in the simulated genomes. This is reflected in the bulk of the cloud of data points, as well as the mean cumulative over all simulations for a given *s*, falling above the diagonal line in the graph. The number of points above, on and below the diagonal are given explicitly in a box at the center of each diagram. There is one clear exception, the yeast with the most highly rearranged (in Figure 1C) and highly reduced (Figure 1B) genome, with the fewest AUs (Figure 1D), namely the atypically asexual [19]* C. glabrata*, which manifests a random pattern, but this does not detract from the clear tendency for concentration of single copy genes in the four other yeast genomes. The particularly striking case of *Arabidopsis *has previously been characterized in some detail [4].

### Control on the simulation experiment

**Figure 5:**
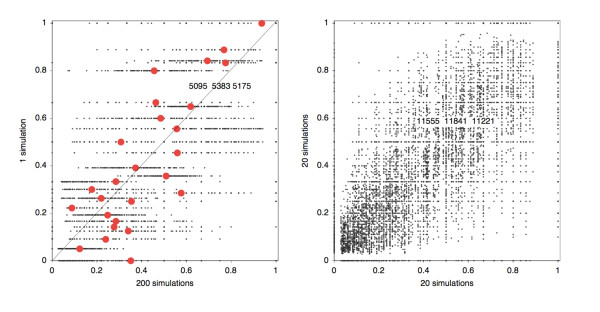
**Confirmation that random genomes cannot produce the effects in Figure 4**. (A) One random genome compared to 200 simulations, as in Figure 4. (B) Twenty random genomes compared to each of 20 other random genomes.

Figure 5 shows typical results of using random genomes instead of the real genomes in Figure 4. It can be seen that there is no evidence here of the "concentration" or "biased fractionation" effect apparent in almost all the genomes in Figure 4.

**Figure 6:**
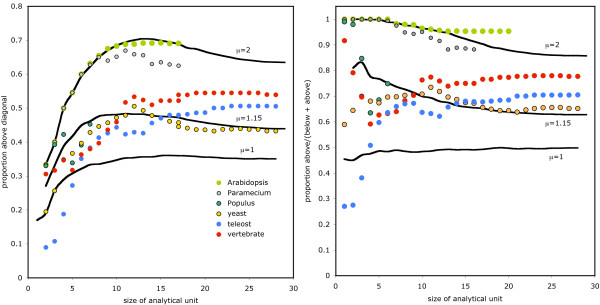
**Proportion of AUs above diagonal in Figure 4, separated by size *s***. (A) Proportion compared to geometric deletion models with *μ *= 2; *μ *= 1.15 and *μ *= 1, the latter representing deletion of one gene at a time. (B) Proportion of AUs above the diagonal when those on the diagonal are ignored.

To analyze these tendencies in more detail, in particular whether the plots in Figure 4 can help us distinguish between the random DNA fragment excision and one-gene-at-a-time explanations, we decomposed the boxed triples of the number of dots above, on and below the diagonal according to the length *s *of the AU. As a summary statistic we calculated the proportion of dots above the diagonal for each *s*. These are plotted in Figure 6, both the proportion of all dots, above, below and on the diagonal (Figure 6A) and the proportion of off-diagonal dots, i.e., above or below the diagonal only (Figure 6B). For those WGD with more than one descendant in our sample, we averaged the scores over all the descendants.

The random DNA fragment excision hypothesis is most simply modeled by a one-parameter exponential (or geometric) decline in deleted fragment length, Thus we carried out a series of simulations with *n *= 12,000, *s *= 10,000 and *d *= 500. For each random deletion event, we picked a random number ≥ 1 of adjacent deletions according to a geometric distribution with mean *μ*. (Note that these geometric distributions are defined over the positive integers *s*, since it is meaningless to consider runs of *s *deleted genes where *s *= 0, whereas the geometric distributions in Figure 3 are defined over the non-negative integers, since *s *= 0 corresponds to the important case of conservation of adjacent paralogy pairs.) The results of three of these experiments (200 runs each), with *μ *= 1.0, *μ *= 1.15 and *μ *= 2 are also depicted in Figure 6.

Two main patterns emerge in both Figure 6A and Figure 6B. First, genomes descended from the "young" WGD events affecting *Paramecium *and, especially, *Arabidopsis *closely follow the geometric simulation with mean *μ *= 2. The other genomes are not well modeled by any geometric distribution. The example shown, with *μ *= 1.15, overpredicts the proportion of small AUs with a high degree of fractionation and underpredicts the proportion of large AUs with this property. The experiment with *μ *= 1 confirms that smaller *μ *worsen the fit with the long AU data while the *μ *= 2 results confirm that larger *μ *worsen the overprediction.

If we hypothesize that the "young" pattern of *Paramecium *and *Arabidopsis *resolves itself over hundreds of millions of years into the pattern displayed by yeast, vertebrates and teleosts, how can this change be interpreted? First, as paralog reduction proceeds, AUs are enlarged by the loss of internal "pillars" at their borders and the merger of of smaller AUs. Moreover, the largest number of AUs have length *s *= 1, and are hence not pertinent to the concentrated-balanced distribution of single-copy genes and so do not play a role in Figures 4 and 6. Many of these, however, will grow into larger AUs. All these processes are combinatorially more likely to attenuate fractionation than to increase it. However, changes in fractionation by the addition of one or a few new single-copy genes will not change the position of the dot corresponding to a long AU in Figure 4 as much as it will a short AU. Thus the greater proportional loss of dots above the diagonal for smaller AU in the case of the more ancient WGD.

The problem of runs of single-copy genes can then be properly situated in the context of analytical units consisting of originally duplicated segments on two chromosomes instead of one, uninterrupted by rearrangements since the WGD, and rephrased in terms of the concentration of single copies on either one of these chromosomes. Here we can reject the random model in favor of a universal tendency towards a substantial degree of concentration. This may well be due to universal biophysical or *in vitro *properties governing DNA fragmentation. This explanation, however, would be difficult to reconcile with the wide range of gene sizes and intergenic spacing in the lineages we have studied. Alternative plausible explanations for the tendency to produce long runs of single-copy genes on individual chromosomes within the AU are a widespread neighborhood selection effect, possibly at the transcriptional level, perhaps involving co-regulation or common regulatory elements [20], dosage sensitivity [3], or differential epigenetic marking [4,21].

Table 1 contains a comparison of the 15 genomes with respect to WGD date, numbers of genes entering the analysis, halving distance, AUs, geometric parameter, and simulation results in Figure 4.

**Table 1:** Summary statistics for 15 genomes. *t*: millions of years since WGD. *n*: number of genes. *m*: number of single copy genes. *d*: halving distance.

genome	*t*	*n*	*m*	*d*	AU			above/on/below	*p*
*S. cerevisiae*	150	5616	4498	135	313	0.89	0.24	6598/5796/4178	0.17
*C. glabrata*	150	5180	4382	252	112	0.92	0.63	2119/3563/2667	0.18
*V. polyspora*	150	5112	4164	202	180	0.90	0.43	6354/4298/2624	0.21
*S. bayanus*	150	5857	4773	186	276	0.90	0.34	7300/5556/4007	0.17
*N. castelli*	150	5213	4053	221	251	0.88	0.38	5159/4412/2876	0.21

*Paramecium*	20	38626	14576	214	5276	0.55	0.02	6763/3155/904	0.49

*Populus*	70	20082	7228	2600	1020	0.53	0.41	1615/1412/542	0.62

*Arabidopsis*	50	25655	13267	2701	718	0.68	0.43	6626/2645/327	0.29

fugu	350	14251	12653	374	222	0.941	0.468	6709/3735/3245	0.27
medaka	350	14564	13352	362	143	0.957	0.597	8958/4838/2708	0.24
stickleback	350	16726	14876	519	240	0.941	0.561	871/5178/4110	0.27
*tetraodon*	350	17120	16088	310	129	0.969	0.601	13279/6184/3360	0.18

chicken	450	10077	8495	686	49	0.915	0.867	2776/2259/1262	0.46
opossum	450	13339	11589	751	69	0.93	0.858	8124/4182/1570	0.27
human	450	13828	12144	673	88	0.935	0.799	7484/3383/1761	0.29

AU (m > 0): analytical units, excluding adjacent pairs of paralogs.  proportion of paralogs lost.  normalized having distance (d depends only on the configuration of the  pairs of duplicate genes). "above/on/below": number of real-simulated comparisons above, on and below the diagonal, with "above" indicating single copy concentration. p: geometric parameter of the AU distribution (Figure 3)

## Conclusions

We decomposed the question of how WGD paralogs are deleted into two problems, one of the random scattering of reduced pairs across the genome, and the other of the concentration of neighboring conserved paralogs on one chromosome or another. For the first question, we cannot reject random gene-by gene loss.

Nor can we rule out the elimination of geometrically-distributed, random length, DNA fragments. In the latter model, if the segments eliminated are generally smaller than one gene, this mechanism becomes indistinguishable from other processes of single gene inactivation, in any case. We observed a high rate of retention of two or more adjacent paralogs pairs (AUs with *s *= 0) in all genomes except yeast, an effect which may be concentrated in certain functional classes of genes [21]. This observation may be explicable in the same terms as the concentration of the single-copy genes in an AU on one of the two chromosomes, in terms of the co-regulation of dosage sensitive genes [3]. Our results confirm those previously reported for *Arabidopsis*, although the effect is particular salient in this genome.

If the pattern of paralogy reduction inferable from Figure 3 is consistent with independent gene-by-gene reduction, how can we reconcile this with the geometrically distributed *s*-gene loss events in Figure 6? For the older WGD, there is little contradiction, since the parameter of the geometric distribution involved is so close to 1.0 that it would be difficult to distinguish between the two models on the basis of the the AU data. In any case our results in Figure 6 strongly suggest that ongoing selective processes other than paralogy reduction by either DNA elimination or gene-by-gene inactivation are responsible for the current fractionation bias in the descendants of the old events.

For the *Paramecium *and *Arabidopsis *data, which are consistent with the geometric distribution of excised fragment lengths with *μ *= 2, we must remember that half of the fragments under this model will be of length 1. Since an AU with *s *= 1 can only be fractionated one way, this major segment of the data is not considered in Figure 4, 5 or 6, but it is a prominent feature of Figure 3. Moreover, *s *for *Paramecium *and *Arabidopsis *takes on small values compared to the descendants of old WGDs. This means that most AU are constructed from one or two excision events, so that the tendency apparent in Figure 6 is not apparent in any distortion of the geometric distribution in Figure 3.

In general terms then, after the duplication, paralog reduction events occur at a regular rate, affecting random locations across the genome. Certain regions, containing two or more pairs of paralogs adjacent on two chromosomes, resist this reduction over long periods of time. A large proportion of the reduction events affect single genes, so that a paralog pair loses one of its members, with either copy being equally likely to disappear, through pseudogenization preceded possibly by suppression of transcription or other silencing mechanism, or through actual deletion of all or most the exonic DNA from the gene. The latter process may extend to the deletion of two, three or more adjacent genes in a single reduction event, though this is visible only in young WGDs, leaving a run of singe-copy genes on only one of the two chromosomes containing the original paralog pairs. This contrasts with the former process, pseudogenization one gene at a time, which is more likely to distribute the surviving members of duplicate pairs to the two chromosomes at random.

After hundreds of millions of years, neighboring single-copy regions merge to become longer, as the resistant paralog pairs are either reduced or diverge functionally, and single-copy regions are disrupted by genome rearrangements, so that it becomes difficult to discern the pattern of paralog reduction using single-copy region statistics, such as those calculated from our AU data.

Our results (Figure 1B) add credibility to observations [21] that gene loss proceeds more rapidly initially and then levels off, but also suggest a universal pattern of genome collapse summarized by the patterns in Figures 1, 3 and 4 rather than a diversity of responses to WGD in different evolutionary lineages. In particular, different lineages deriving from the same WGD act remarkably similarly as replicates of the same evolutionary "experiment" over hundreds of millions of years.

## Methods

### Constructing the analytical units (AU)

An AU is composed of two segments of the form *p*_1_, *s*,..., *s*, *q*_1 _on one chromosome and *p*_2_, *t*,..., *t*, *q*_2 _on another chromosome (or elsewhere on the same chromosome), where *p*_1 _and *p*_2 _are paralogs dating from the WGD, and so are *q*_1 _and *q*_2_, and the *s *and *t *are single-copy genes in the genome. In addition *p*_1 _and *p*_2 _must have the same reading direction, and *q*_1 _and *q*_2 _must have the same reading direction. Alternatively, the AU can be of form *p*_1_, *s*,..., *s*, *q*_1 _on one chromosome and *q*_2_, *t*,..., *t*, *p*_2 _where *p*_1 _and *p*_2 _have opposite reading directions, as do *q*_1 _and *q*_2_.

### Data

Because our method is based on gene order, and this data is available for few genomes, not all available in a single database, we accessed a number of resources.

#### Yeast, Paramecium, Arabidopsis

For the five yeast genomes, all the data on gene order and paralogy, specifically that paralogy due to the WGD event, is explicitly detailed on the Yeast Genome Browser [10,22].

For *Paramecium*, all the data on gene order and paralogy, specifically that paralogy due to the most recent WGD event, is found in the supplemental materials to reference [9]. For *Arabidopsis*, all the data on gene order and paralogy, specifically that paralogy due to the most recent WGD event, is found in the supplemental materials to reference [12].

#### Poplar

Annotations for the *Populus *genome were obtained from the database maintained by the U.S. Department of Energy's Joint Genome Institute [11]. An all-by-all BLASTP search was run on all *Populus *protein coding genes, and orthoMCL [23] was used to construct gene families. This work was carried out by P. Kerr Wall in connection with the research described in reference [18].

#### Higher vertebrates

Chicken (*Gallus gallus*), opossum (*Monodelphis domestica*) and human (*Homo sapiens*) protein sequence data were retrieved from ENSEMBL ver. 54. We first carried out all-against-all BLASTP between all proteins from a genome (using E-value 1E-5), and between proteins from this genome and outgroups (*Ciona intestinalis, Ciona savignyi, Tetraodon nigroviridis, Danio rerio*).

The paralogous hits give us the initial candidate gene families. We retain those paralogous hits that satisfy two requirements: 1) the paralog alignment is stronger than the alignment of a paralog with the best orthologous *Ciona *protein (*intestinalis *or *savignyi*), and 2) the paralog alignment is weaker than any alignment with the best orthologous fish protein (*nigroviridis *or *rerio*). This step eliminates duplicate genes that arose after the most recent common vertebrate WGD [15,24].

We filter away hits with alignment score < 140 bits in an effort to retain only duplicates produced by the later of the two vertebrate WGDs.

Singleton genes used for our analysis were also verified with an outgroup by checking that the gene has a correspondence in both a fish genome and a *Ciona *genome.

#### Teleosts

We performed all-against-all BLASTP between all proteins in the fish genomes (medaka, stickleback, fugu, *nigroviridis*). The complication here is that the ray-finned fish have undergone both the two common WGD events and the teleost WGD. We know, however, that the distribution of protein identity scores from the teleost WGD is shifted to distinctly higher values compared to the earlier vertebrate WGDs [13]. We thus use best-reciprocal hit (BRH) to separate out the closest paralog-pairs in each gene family to attribute to the teleost WGD.

### Preprocessing

Aside from the filtering of duplicates discussed above, two additional difficulties were encountered as we prepared the data for the simulations necessary to assess concentrations of single copies in AUs. One derived from incomplete assembly of many of the genomes, resulting in many short contigs. To avoid reproducing simulated genomes having this defect, without distorting at the same time the numbers of duplicates and single-copy genes in the genome, we simply discarded contigs unless they contained either at least two duplicated genes or ten single-copy genes.

In the poplar genome, because of the numerous gene families, we estimated the tetraploid ancestor and then created the simulated genomes by initially removing all members of multigene families. We restored these genes on a random basis before constructing the AUs in the simulated genomes.

### WGD dates

We used dates suggested in the primary references cited for each genome [10-13,15]. The *Paramecium *date is speculative, based on protein identity scores [9] compared to human-mouse divergence scores, with allowance made for generation-time differences. The dates enter our analysis only in Figure 1, and all that is really necessary to illustrate the trends depicted is a rank ordering of the dates.

## Authors' contributions

DS, CZ and QZ formulated the problem, carried out the data analysis and simulations, and wrote the paper. All authors read and approved the final manuscript.
